# Adverse psychiatric effects of psychedelic drugs: a systematic review of case reports

**DOI:** 10.1017/S0033291724002496

**Published:** 2024-11

**Authors:** B. Yildirim, S. S. Sahin, A. Gee, S. Jauhar, J. Rucker, P. Salgado-Pineda, E. Pomarol-Clotet, P. McKenna

**Affiliations:** 1 FIDMAG Hermanas Hospitalarias Research Foundation and CIBERSAM, ISCIII, Barcelona, Spain; 2 Department of Psychiatry, Bakırkoy Mazhar Osman Mental Health and Neurological Diseases Education and Research Hospital, Istanbul, Türkiye; 3 Department of Psychological Medicine, Institute of Psychiatry, Psychology and Neuroscience, King's College London, London, UK; 4 South London & Maudsley NHS Foundation Trust, Bethlem Royal Hospital, Kent, UK

**Keywords:** adverse effects, affective disorder, case reports, flashbacks, hallucinogen persisting perception disorder, hallucinogens, psychedelic drugs, psychosis, systematic review

## Abstract

**Background:**

Psychedelic drugs are a focus of interest in the treatment of depression and other disorders but there are longstanding concerns about possible adverse psychiatric consequences. Because the relevant literature is largely informal, the seriousness of these risks is difficult to evaluate.

**Methods:**

Searches were made for case reports of schizophrenia-spectrum, affective or other psychiatric disorders after use of psychedelic drugs. Case reports of flashbacks were also searched for. Individuals with recent use of other drugs (apart from cannabis and alcohol) and/or a previous history of major psychiatric disorder were excluded. Symptoms were tabulated using the Syndrome Check List of the Present State Examination (PSE-9).

**Results:**

We found 17 case reports of schizophrenia spectrum disorder, 17 of affective disorder (depression, mania, or both), 3 cases of anxiety, 1 of depersonalization, and 1 of unclassifiable illness. The states could develop after a single use of the drug (5/17 schizophrenia; 6/17 affective disorder), and duration was highly variable. Recovery was the rule in cases of affective disorder but not in schizophrenia spectrum disorder. Twelve of 29 cases of flashbacks showed psychiatric symptomatology definitely outlasting the attacks, mainly anxiety (5 cases) and depression (8 cases). Flashback symptoms resolved within twelve months in approximately half of the cases but in a few persisted for years.

**Conclusions:**

Reliable descriptions of schizophrenia spectrum disorder and major affective disorder after psychedelic drug use disorder exist but are relatively uncommon. Flashbacks are sometimes but not always associated with psychiatric symptomatology, mainly anxiety or depression.

## Introduction

After early interest, followed by criminalization and the halting of almost all human research (Lieberman, 2021; Rucker, Iliff, & Nutt, 2018), the potential therapeutic effects of psychedelic drugs are once again a focus of worldwide attention. Four recent trials (Carhart-Harris et al., 2021; Goodwin et al., 2022; Raison et al., 2023; von Rotz et al., 2023) (one of them large, *N* = 233; Goodwin et al., 2022) have found evidence for psilocybin's effectiveness in major depressive disorder, leading to the FDA designating it a ‘breakthrough therapy’ for treatment-resistant patients with the disorder. A 2020 review (Reiff et al., 2020) also identified four contemporary trials of psychedelics (three of psilocybin and one of LSD) for anxiety and/or depression associated with life-threatening disease, with another published since then (Holze, Gasser, Muller, Dolder, & Liechti, 2023). The same review found two trials of ayahuasca, a hallucinogenic preparation used in South American shamanic ceremonies whose active ingredient is dimethyltryptamine (DMT), for major depression. Two double-blind placebo-controlled trials of psilocybin for obsessive-compulsive disorder are currently underway (Ching et al., 2023a, 2023b).

The therapeutic benefits of psychedelics may come at a price, however. After initially being considered infrequent (Cohen, 1960), reports of adverse psychiatric effects increased dramatically after recreational use of LSD became widespread (Schwarz, 1968; Smart & Bateman, 1967). Prominent among these were schizophrenia: reviewing the literature in 1979, Klawans, Tanner, and Goetz (1979) concluded that there was evidence for a chronic psychosis associated with use of LSD, which usually occurred in individuals who had taken it 50 or more times and whose symptoms included ‘paranoid ideation, prominent use of denial and projection, poorly organized and bizarre thoughts, intermittent visual hallucinations, shallow affect, withdrawn behavior, and loss of long-term memory.’ The presentation was considered comparable to chronic undifferentiated schizophrenia, with the exception that interpersonal functioning was preserved in some cases.

In contrast, affective disorder as a complication of psychedelic drug use has historically attracted little attention. After analyzing case reports up to 1967, Smart and Bateman (1967) concluded that purely depressive states appeared to be rare, and they found only one case of a ‘motor excitatory state’. Depression was not mentioned at all in a later review (Strassman, 1984). In the view of Cohen (1985), an author who wrote extensively on LSD, manic and depressive episodes could be seen, although less frequently than schizophrenia-like states. This author also noted the occurrence of other disorders characterized by anxiety, panic, or confusion, as well as dissociative states and states characterized by depersonalization.

The explosion of use of LSD and other psychedelic drugs also led to recognition of a further adverse psychiatric effect: flashbacks, spontaneous recurrences of aspects of the psychedelic experience, particularly the perceptual changes. This phenomenon had occasionally been noted by early authors after use of LSD and mescaline therapeutically or experimentally (Cooper, 1955; Harley-Mason, Laird, & Smythies, 1958; Sandison & Whitelaw, 1957). However, credit for its formal description is probably due to Cohen and Ditman (1963): they described two regular LSD users who saw faces or skulls moving on walls, and a child who accidentally took LSD and a week later experienced movements on the TV screen and ‘light halos’ with his eyes closed; in all three cases the experience was accompanied by anxiety. Further descriptions followed (e.g. Cohen, 1966; Horowitz, 1970; Rosenthal, 1964; Smart & Bateman, 1967), and in 1987 the term hallucinogen persisting perception disorder (HPPD) was introduced in DSM-III-R. This included a requirement for flashback symptoms to be associated with clinically significant distress or impairment in social, occupational, or other areas of functioning. The criteria for the disorder remain essentially unchanged in DSM-5.

Notwithstanding an extensive literature, determining whether and to what extent psychedelic drugs can cause psychosis or other psychiatric disorders faces difficulties. Firstly, many of the relevant studies date from the 1960s and 1970s, when the concept of schizophrenia was looser than it is today, especially in the United States, where the use of psychodynamic terminology like ego-fragmentation and projection also complicates interpretation. Secondly, psychedelic users who develop psychiatric disorders will likely have used other drugs as well, and some of them may have had pre-existing psychiatric disorders. Similar problems apply to the literature on flashbacks/HPPD, which remains a generally under-researched topic.

One strategy for establishing how far psychedelics are associated with development of psychiatric disorders is by means of systematic review of case reports. Such an approach can help define the range of disorders seen and, in the case of flashbacks/HPPD, answer questions about the nature of perceptual changes that characterize them, as well as potentially shedding light on how far they are distressing and/or associated with impaired functioning. In this study we searched the worldwide case report literature on psychiatric complications of use of psychedelic drugs, excluding individuals who showed concurrent use of other drugs (apart from cannabis and alcohol) and those who had a previous history of major psychiatric disorder, and formally tabulating the symptoms described.

## Method

We searched PubMed (which includes MEDLINE), Embase and psycINFO for articles reporting individual cases of any kind of adverse psychiatric event in the context of use of psychedelic drugs. Drugs considered as psychedelics included LSD, lysergic acid amide (morning glory seeds and Hawaiian baby woodrose seeds), mescaline/peyote, psilocybin, dimethyltryptamine (DMT)/ayahuasca and the amphetamine-related drugs, 2,5-dimethoxy-amphetamine (STP or DOM), and 2,5-dimethoxy-bromoamphetamine (brolamfetamine, bromo-DMA). These drugs all have 5HT2A receptor partial agonism as their principal mode of action, the property now recognized as responsible for conferring psychedelic effects (Nichols, 2016). Bufotenine was also included in the search although there is some doubt about whether its effects are truly psychedelic and to what extent it exhibits 5HT2A agonism (Lyttle, Goldstein, & Gartz, 1996) (in the event no relevant articles were found for this drug). Ibogaine and MDMA were not included. Ibogaine's experiential effects do not closely resemble those of classical psychedelic drugs and its mechanism of action appears to be only minimally related to activity at 5HT2A receptors (Alper, 2001; Mash, 2023). The experiential effects of MDMA are also different from those of psychedelic drugs and its major pharmacological effect is stimulation of serotonin release (Iversen, 2008).

Search terms included hallucinogens, individual drug names, and terms relating to adverse psychiatric effects. The databases were searched up to 21/06/23 and 26/06/23 (for two searches in PubMed, with and without a ‘case report’ filter) and 30/6/23 (for PsycINFO and Embase). No start dates were specified, but we did not formally consider articles published before 1950, as it became clear these only described the psychedelic experience, not complications of it. Four of the authors (BY, SS, AG, PJM), working in groups of two or three, examined the titles and abstracts of publications identified by the electronic search and obtained full versions of all that were considered potentially relevant. We hand-searched reference lists of case reports, studies and review articles, and also checked a number of books that were found. There were no restrictions on language. We considered reports in adolescents and adults with no upper age limit; cases in children were also considered (one such case was included). Details of the searches are given in the Supplementary Information and also in Open Science Framework, where the protocol is registered (https://osf.io/k47g6).

To be included, we required publications to (a) report one or more cases, and (b) give sufficient description of symptoms to allow tabulation using a structured instrument (see below); articles providing only summarized information (e.g. conference abstracts) were not included. Cases were excluded if there was a history of major mental illness (schizophrenia, major depression, bipolar disorder) prior to the index episode, or if there was presence of neurological or other disease affecting brain function, or if the symptoms did not outlast the period of intoxication (defined as 24 hours after taking the drug or 3 days in the case of STP/DOM). We excluded case reports of patients who had used other drugs within a year of the episode, with two exceptions, cannabis and non-dependent use of alcohol.

Symptoms were tabulated using the Syndrome Check-List (SCL) of the Present State Examination (PSE), 9th edition (Wing, Cooper, & Sartorius, 1974), which was developed to be applied to non-interview information such as casenotes. Typically, one or more symptoms contributing to a syndrome need to be rated for the syndrome to be considered present. Following the checklist guidelines, we did not rate simple statements that the patient had delusions or hallucinations, but required description of the content. Similarly, the SCL syndromes *incoherent speech* and *depersonalization* (which covers both depersonalization and derealization) were only rated if there was some additional description to make it clear that the relevant symptoms were being described.

The SCL rates depression under three syndromes, *simple depression* (inefficient thinking, depressed mood, hopelessness, suicidal plans or acts, depression on examination), *special features of depression* (self-depreciation, guilty ideas of reference, guilt, dulled perception, lost affect) and *other symptoms of depression* (morning depression, loss of appetite, early waking, loss of libido, premenstrual exacerbation). An additional syndrome, *loss of interest and concentration* is also relevant to depression, and there is a psychotic syndrome, *depressive delusions and hallucinations*. Manic symptoms are rated under *hypomania*, comprising subjective euphoria, ideomotor pressure, grandiose ideas and actions, hypomanic affect and hypomanic content of speech. Perceptual changes of the kind seen in flashbacks/HPPD were rated under the SCL item *non-specific psychosis*, which contains items for heightened perception, changed perception and minor visual, auditory and other hallucinations, among other symptoms; each case's individual clinical features were also recorded.

Extraction and tabulation of symptom data was carried out by PJM, who is trained in the use of the PSE. Where there were doubts about inclusion, the PSE glossary of symptoms was consulted.

## Results

**Figure 1.**
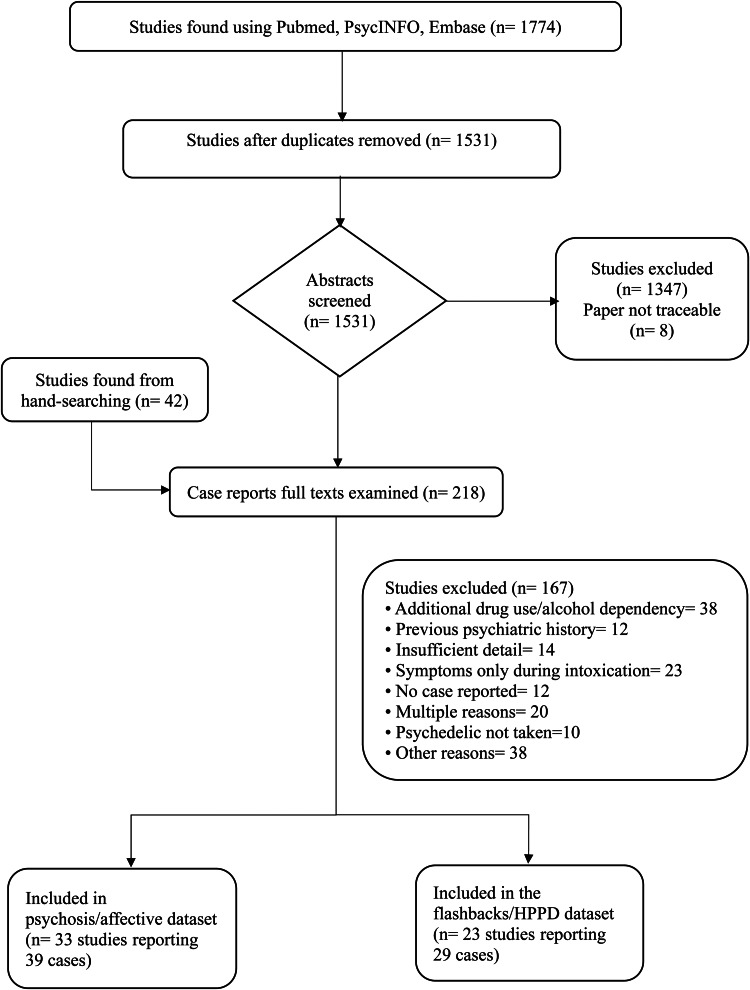
Flow chart of the results of the search process.

A flow chart is shown in Fig. 1. Thirty-nine case reports of psychiatric disorders (other than flashbacks/HPPD) from 33 publications were finally included and 29 case reports of flashbacks/HPPD from 23 publications.

### Schizophrenia spectrum disorder

There were 17 case reports of schizophrenia spectrum psychosis; the findings are summarized in Table 1. The psychedelic drugs taken were LSD (9 cases), lysergic acid amide (3 cases, including one who took both LSD and lysergic acid amide), ayahuasca (2 cases), mescaline (1 case) psilocybin (1 case) and bromo-DMA (1 case). In 5 cases the state developed after a single dose was taken, in 2 after two doses were taken and in 4 after less than ten doses (including one reported as ‘several times’). The remaining cases were regular users over months or years. Cannabis use was commented on in seven cases and alcohol use in one. As can be seen from Table 1, psychotic symptoms included delusions (referential, persecutory, religious, sexual and fantastic grandiose and guilty), hallucinations (auditory and visual), formal thought disorder, and affective flattening and incongruity; three cases had catatonic symptoms. There was evidence of a mood component to the illness in several cases: in 4 the state was associated with depressed mood and in 1 more there was prominent guilt and rumination; 3 more cases showed overactivity.

**Table 1.** PSE syndromes in 17 case reports of psychosis

Case	Age	Sex	Drug	No of times taken	Other drug use	PSE syndromes	Other symptoms/symptoms without examples	Comments
1. Cohen and Ditman (1963) Case 1	36	f	LSD	1	None	Incoherent speech Grandiose and religious delusions Overactivity	–	–
2. Cohen (1964)	24	m	Lysergic acid amide	1	No details	Delusions of reference Anxiety	‘Loose associations’ Ringing in ears	Committed suicide a week after onset of symptoms
3. Fink, Goldman, and Lyons (1966) Case 2	21	m	Lysergic acid amide	Not stated	Mescaline	Simple depression Grandiose and religious delusions Overactivity	Incoherent speech ?Auditory and visual hallucinations (no description) Sexual and fantastic delusions possibly only during intoxication) Mood lability	–
4. Flach (1967)	20	m	Lysergic acid amide	2	Cannabis	Incoherent speech Anxiety Auditory hallucinations Delusions of persecution Grandiose and religious delusions Sexual and fantastic delusions Overactivity Agitation Irritability	?Visual hallucinations (no description) Lability of affect ?Catatonic syndrome	–
5. Rosenberg and Eldred (1968) Case 4	19	f	LSD	6	No details	Delusions of persecution	–	–
6. Jørgensen (1968) Case 4	21	m	LSD + Lysergic acid amide	8 times	Cannabis	Anxiety Delusions of persecution	Incongruous affect Auditory hallucinations (no description) ‘Thoughts jumping’	–
7. Glass and Bowers (1970) Case 2	21	m	LSD	>200	No details	Incoherent speech Blunted affect Delusions of persecution	?Non-specific psychosis (bizarre appearance) ‘Bizarre ideas of reference’ Visual hallucinations NOS	–
8. Glass and Bowers (1970) Case 4	21	m	LSD	>250	No details	Incoherent speech Blunted affect	?Non-specific psychosis (bizarre appearance) ‘Paranoid delusions about his wife’	–
9. Rosen and Hoffman (1972) Case 2	22	f	LSD	>6	Cannabis	Religious delusions Special features of depression (guilt)	Withdrawal Guilty ruminations Self-starvation Inappropriate behaviour	Acted on delusions by removing her eye.
10. Hyde, Glancy, Omerod, Hall, and Taylor (1978)	20	m	Psilocybin	Several times	No details	Catatonic syndrome Incoherent speech Delusions of reference Agitation	–	Illness was short and followed very large dose of mushrooms (50–60), so could construe it as due to acute effects of drug
11. Braconnier and Schmit (1979) Case 1	16	m	LSD	Around 20	Cannabis	Depressive delusions and hallucinations Simple depression General anxiety Delusions of persecution Sexual and fantastic delusions Visual hallucinations Non-specific psychosis Social unease	?Auditory hallucinations (no description)	Not completely clear whether experienced visual hallucinations or illusions based on real perception.
12. Braconnier and Schmit (1979) Case 3	16	m	LSD	Not stated	Cannabis	General anxiety Auditory hallucinations Affective flattening Sexual and fantastic delusions Non-specific psychosis	Depersonalization (may have been delusional)	–
13. Metzner (1979)	22	m	LSD	4 or 5	No details	Catatonic syndrome Incoherence of speech	Incongruent affect	
14. Lu, Woofter, and Escalona (2004)	54	m	Mescaline (peyote)	1	Alcohol in past	Simple depression Auditory hallucinations Delusions of persecution Visual hallucinations	–	Alcohol abuse and post-traumatic stress disorder ‘in remission for 20 years’.
15. Neyra-Ontaneda (2017)	40	m	DMT (ayahuasca)	1	Alcohol	Auditory hallucinations Delusions of persecution Irritability	?Non-specific psychosis (suspiciousness)	‘Probable convulsion’ 4 years earlier.
16. Scarella and Teslyar (2019)	24	f	Bromo-DMA	1	Cannabis	Catatonic syndrome Depressive delusions and hallucinations Delusions of persecution Agitation	?Visual hallucinations (no description)	Possible periods of confusion x2 during course of illness.
17. Neu (2020)	27	m	DMT (ayahuasca)	2	Cannabis	Simple depression Anxiety ?Nuclear syndrome (partially held delusions of control) Delusions of persecution	–	–

The duration of the state was reported in 15 cases and ranged from a few days to 2 weeks in six cases (5, 10, 14, 15, 16, 17); from 3 to 6 weeks in two (12, 13); and from 2 to 6 months (or longer, given the reported lack of improvement) in seven (1, 3, 4, 6, 8, 9, 11). With respect to outcome, 11 cases recovered or were left with only minor symptoms (3, 4, 5, 9, 10, 11, 13, 14, 15, 16, 17). Three cases were described as improved (1, 6, 12) and two as unimproved (7, 8). A further patient committed suicide while acutely ill (case 2).

### Affective disorder

There were 17 cases of affective disorder: 11 of depression, 3 of mania, and 3 of depression and mania in the same or different episodes (see Table 2). A majority of cases (12) occurred in the context of use of LSD, but there were also cases associated with use of ayahuasca (2), lysergic acid amide (1), mescaline (1, with some doubt about whether this was the drug taken) and psilocybin (1). Six cases developed after only a single dose was taken, 1 after two doses, 1 after three doses, 1 after four doses, 2 after five doses, and the remainder after multiple or unknown numbers of doses. Psychotic symptoms were present in seven cases. Concurrent cannabis use was noted in eight cases. Alcohol use was commented on in three cases with one more patient having drunk heavily up to a year previously.

**Table 2.** PSE syndromes in 17 cases of affective disorder

Case	Age	Sex	Drug	No of times taken	Other drug use	PSE syndromes	Other symptoms/symptoms without examples	Comments
*Depression*								
18. Hoffer and Callbeck (1960)	35	f	LSD	3	No details	Special features of depression (lost emotions) Lack of energy Loss of interest and concentration Other symptoms of depression	Found it difficult to use words, as they were loaded with meaning For first three days, curtains had a ‘tapestry’ appearance. Various physical symptoms, esp. feeling cold	Penicillamine administered with LSD (given therapeutically). Received several doses of methedrine, but only after illness started.
19. Cohen and Ditman (1963) Case 6	Not given	m	LSD	1	Implies no	Simple depression Hypochondriasis	–	–
20. Jørgensen (1968) Case 2	21	f	LSD	4	Cannabis	Simple depression Irritability	–	Patient improved but later committed suicide.
21. Hatrick and Dewhurst (1970) Case 1	19	f	LSD	1	Implies no	Simple depression Non-specific psychosis Depersonalization Special features of depression (self-depreciation, guilt)	‘Tangential thought disorder’	Said to have become schizophrenic after depression improved, but only symptoms were heightened and distorted perception, depersonalization/derealization and possible formal thought disorder.
22. Hatrick and Dewhurst (1970) Case 2	21	f	LSD	1	Implies no	Simple depression Auditory hallucinations Special features of depression (guilt)	–	Auditory hallucinations described as being ‘of a persecutory nature’.
23. Linken (1971) Case 3	Not given	m	LSD	Not stated	No details	Anxiety Simple depression	Continuous ruminations over abstruse questions such as the meaning of life, religion and the universe	Not clear if ruminations were obsessional in nature.
24. Fookes (1972)	27	m	LSD	2	Implies no	Simple depression Special features of depression (guilt)	Saw poster with mirror writing on it just before a suicide attempt	Had idea that God had tired of him because he had done something very wrong Made two serious suicide attempts.
25. Glass (1973) Case 1	17	f	Mescaline	5	Cannabis	Depressive delusions and hallucinations Delusions of reference	Depersonalization Hallucinations (no description)	Doubt expressed over whether drug taken was actually mescaline.
26. Kaminer and Hrecznyj (1991)	15	m	LSD	Around 180	Cannabis Alcohol	Simple depression Other symptoms of depression	Non-specific psychosis (unintelligible whispers, visual distortions)	Experienced constant flashback phenomena (colored dot patterns, trailing, halos round objects.)
27. Markel, Lee, Holmes, and Domino (1994) Case 1	18	f	LSD	Multiple (weekly)	Cannabis Alcohol	General anxiety Simple depression Loss of interest and concentration Other symptoms of depression	Non-specific psychosis (heightened and distorted perception) in context of flashbacks only	Also experienced flashbacks.
28. Dos Santos (2011)	21	m	Ayahuasca	Multiple	Other hallucinogens Cannabis	Depressive delusions and hallucinations Delusions of persecution	–	Recovered, then symptoms recurred a year later taking ayahuasca again.
*Mania/bipolar*								
29. Opitz (1963) Case 2	47	m	LSD	5	Painkillers (aspirin, codeine, caffeine)	Hypomania Irritability Simple depression Anxiety	Lability of mood	Hypomanic 2 1/2 months then depressed 2 months. Before treatment had anxiety, hypochondriacal concerns and interpersonal difficulties. Alcohol abuse in past.
30. Rosenberg and Eldred (1968) Case 3	22	m	LSD	Not stated	Cannabis	Depressive delusions and hallucinations Simple depression Grandiose and religious delusions Special features of depression	–	Predominantly depressive episode, but towards end developed fixed belief he had cured another patient Used alcohol until a year previously.
31. Lake, Stirba, Kinneman, Carlson, and Holloway, (1981)	31	m	LSD	1	Alcohol	Hypomania Religious delusions Irritability	–	–
32. Sobanski et al. (2021)	17	f	Lysergic acid amide	1	No details	Hypomania General anxiety Special features of depression (guilt) Poor concentration	Incoherent speech without examples Ideas of reference, implied to be delusional	Hypomanic then became depressed. Guilt may have been delusional.
33. Alcantarilla, García-Alcarria, and Almonacid (2022)	21	f	Ayahuasca	1	Cannabis	Hypomania Grandiose and religious delusions	Visual distortions	Episode developed in the days following ingestion, and he continued to experience sensory distortions (changes in size and shape of objects, heightened visual and tactile perception).
34. Suleiman, Basu, Belal, and Jacob (2022)	18	m	Psilocybin	No details	Cannabis	Hypomania Overactivity Agitation	Delusions of persecution (no description)	Overactivity severe (‘endless, frenzied motor activity and engaging in inappropriate behaviour’).

There were no cases with brief durations (≤2 weeks). Duration ranged from between 2–3 weeks and 2 months in nine cases (18, 21, 23, 25, 28, 31, 32, 33, 34), and from 3 to 12 months in seven cases (19, 22, 24, 26, 27, 29, 30). One patient committed suicide after making an apparent full recovery following approximately 4 months of illness (case 20). Not including this last case, the outcome was reported as recovery with or without minor symptoms in 13 cases (18, 19, 21, 22, 23, 24, 25, 27, 28, 29, 31, 32, 33), or improvement in 3 cases (26, 30, 34).

Two patients who developed depression also experienced flashbacks, but there was little to suggest that these played a causal role: case 27 had had only four flashbacks and in case 26, flashbacks were only discovered on questioning and appeared to be independent of the mood symptoms.

### Other psychiatric disorders

There were three cases of anxiety disorders precipitated by psychedelic use, two with LSD (McGennis, 1979; Shick & Smith, 1970) and one with psilocybin (Benjamin, 1979). All three cases took the form of generalized anxiety, accompanied by panic attacks in one of them. The state occurred after taking the drug three times, ‘a number of times’ or (probably) many times. There were few details concerning duration or degree of recovery.

There was one case of depersonalization and derealization (Abraham & Mamen, 1996). This developed in an 18-year-old male a week after he took the last of 6–8 doses of LSD. The symptoms developed in association with flashbacks but then became persistent. The state persisted for 20 years, at which point he developed depression and anxiety. Drug treatment brought about only at most minor improvement.

One case could not be classified (Harley-Mason et al., 1958): two weeks after taking mescaline for research purposes, a man had a five-day period of continuously experiencing patterned visual experiences, only this time muddy rather than bright as previously. The state was accompanied by generally dulled perception, unease, loss of appetite and a feeling of physical and mental exhaustion.

### Flashbacks/HPPD

Twenty-nine includable case reports were found. They were mainly reported in association with LSD use (21 cases), with the remaining drugs being psilocybin (2 cases), mescaline (2 cases) and multiple/unspecified psychedelic drugs (4 cases). Cannabis was also used in 9 cases, and alcohol use was recorded in 5.

In 7 cases flashbacks began occurring after only a single dose of the drug was taken, in 1 after two doses were taken, in 2 after use that was sporadic (‘occasional’ or ‘several times’); in the remainder the drug was taken from 6 to 180 times. In over half (15 of 23 cases where information was provided or implied) the symptom resolved within a year after stopping taking psychedelics, with or without treatment. However, cases were also reported with durations of 2, 3, 8, 14, 22, 23 and 25 years.

Details of the features of the flashbacks are given in Table 3. It can be seen that visual afterimages (also referred to as trailing and palinopsia) was the most common perceptual experience, with other elementary visual changes and distortions, such as patterns, flashes of light and color, shimmering and halos round objects occurring less frequently. Unexpectedly, there were descriptions of formed images, including of animals, human figures, and inanimate objects in ten cases. Auditory experiences, ranging from distortions of real sounds to elementary sounds to unintelligible whispers, were described in four cases, and somatosensory disturbances in two. Experiences of unreality and/or depersonalization were reported in 6 cases.

**Table 3.** Flashback phenomena as described in 29 case reports

Phenomenon	Cases reporting	Examples
Flashes of light or color/sparkles/flickering	√√√√√ ^1–5^	Golden translucent patches and golden glitter; stationary shifting points of color.
Moving points of light/darkness/dots	√√√√ ^6–8^	Patterned objects appeared to move; words moving on page; black moving pinpoints.
Shimmering/halos round objects	√√√√√ ^6–8, 10^	Colored halos round objects; red objects having green shimmer around them; light halos with eyes closed.
Distortion of objects/visual illusions	√√√√√√√√√√ ^1, 5, 6, 8, 11–16^	Light breaking up into droplets of color; visual distortions of objects and ‘reliefs’; saw colors that were not there; objects appearing multi-layered; pleasant shifting panels of light; faces changing shape, alteration of own reflection; suddenly feeling as if in a cartoon; alteration of own reflection; flying birds looking like animation; buildings leaning inwards.
Feelings of unreality/depersonalization/derealization	√√√√√√ ^3, 6, 11, 12, 17, 23^	Transient feelings of unreality; derealization not further described; didn't recognize himself in the mirror.
Change in size/distance of objects	√√ ^8, 18^	Altered distance estimation; vehicles appearing to stretch as they went past.
Stationary objects move	√√√ √ ^8, 10, 18, 19^	Patterned objects appearing to move; words moving on page; still objects moving back and forth; movements on TV screen when turned off.
Geometric shapes/spirals/patterns	√√√√√√ ^1, 6, 12, 15, 20^	Seeing colorful geometric forms with eyes closed; seeing spirals that would wrap around his body; patterns in wood; sees round objects with eyes closed.
Afterimages/palinopsia/trailing	√√√√√√√√√√√√√ ^1–7, 12, 23^	–
Images in peripheral visual field	√ ^12^	–
Intensified/distorted visual perception	√√√√√ ^5, 6, 8, 15, 16^	Intimidated by loud colors; vivid colors; colors vivid and unreal; faces changing shape, alteration of own reflection.
Objects pulsating/undulating/wavering	√√√√ ^6, 10, 17, 21^	Everything pulsing and breathing; movement of walls as if breathing; walls moving in and out; pages on books wavering.
Formed visual experiences	√√√√√√√√√√ ^3, 4, 9, 10, 13, 14, 16, 20, 22^	Seeing faces; spontaneous imagery of faces; visual and kinesthetic image of himself crashing through his car window; black and white images of a figure being sucked into a whirlpool; demons gesticulating and poking their tongues out; animals and faces moving on the wall; walls around him would ‘tend to crumble’ and buildings would rise into the air and swallow up the ground; illusions of movies with bodies of water and people in motion; people decomposing in front of her; people transformed into ‘monsters’; image of dragon when flushing toilet; animals and faces moving on the wall.
Auditory perceptual disturbances	√√√√√ ^5, 7, 11, 15, 21^	Distortion of sounds; unintelligible whispers; auditory disturbances with ‘resonance feeling’; rushing sound in ears.
Somatosensory disturbances	√√ √^11, 15, 23^	Feeling of lightness/weightlessness; sinking feeling and paralysis; body image distortion not further specified.
Other	√√√√√	TV spoke to patient^21^; time slowed down^22^; difficulty focusing vision^8^; multicolored patterns that in some way he could also fee ^20^; feeling of impending doom^10^.

1. Kawasaki and Purvin (1996); 2. Lerner, Oyefe, Isaacs, and Sigal (1997); 3. Anderson and O'Malley (1972); 4. Horowitz (1964); 5. Haslacher, Novkovic, Buthut, Heinz, and Soekadar (2022); 6. Abraham and Mamen (1996); 7. Kaminer and Hrecznyj (1991); 8. Noushad, Al Hillawi, Siram, and Arif (2015); 9. Twemlow and Bowen (1979); 10. Cohen and Ditman (1963); 11. Espiard, Lecardeur, Abadie, Halbecq, and Dollfus (2005); 12. Young (1997); 13. Smith and Seymour (1985); 14. Rosenthal (1964); 15. Kleber (1967); 16. Markel et al. (1994); 17. Charre et al. (2020); 18. Lerner and Ran (2015); 19. Harley-Mason et al. (1958); 20. Scott (1971); 21.Skryabin, Vinnikova, Nenastieva, and Alekseyuk (2018); 22. Horowitz (1970); 23. Lerner, Skladman, Kodesh, Sigal, and Shufman (2001).

Twelve of the 29 cases showed associated psychiatric symptomatology that persisted beyond the flashbacks. Anxiety outlasting or occurring outside flashback periods were present in five cases, usually but not always related to fear of having an attack. Eight patients developed persistent depressive symptoms in response to flashbacks. One patient developed hypochondriasis, becoming convinced he had Creutzfeldt-Jakob disease because of his flashback symptoms (Charre, Herrero, Martelli, & Benyamina, 2020). This patient also experienced depersonalization/derealization that probably outlasted the flashbacks. In one case (Lerner & Ran, 2015) the only associated psychiatric symptom was worry.

In a further 6 cases anxiety was only experienced during the flashbacks themselves. In 3 cases it was unclear when the anxiety occurred. One further case (Abraham & Mamen, 1996, also reported in the ‘other psychiatric disorders section) developed persistent depersonalization/derealization with persistent flashbacks after taking LSD 6–8 times; 20 years later he also developed depression and anxiety.

Among the remaining 11 cases no associated psychiatric symptomatology was noted. Three of these cases were in an ophthalmological journal, and the others more gave few details of any kind, leaving open the question of whether psychiatric symptoms were simply not reported.

## Discussion

This systematic review found credible case reports of an association between use of psychedelic drugs and schizophrenia spectrum psychosis, and similar case reports of an association with affective disorder, including both depression and bipolar disorder. Both types of disorder were seen in individuals without a previous psychiatric history and were not, or at least were not wholly, attributable to concurrent use of other drugs, since we excluded cases where there was co-existent substance use apart from alcohol and cannabis.

Our ability to find only 17 case reports of psychosis in individuals who used psychedelic drugs contrasts with a relatively entrenched view in psychiatry and society as a whole that such drugs are strongly associated with this form of psychiatric disorder. For example, several well-known rock musicians who developed schizophrenia are widely considered to be ‘acid casualties’. It also contrasts with a previous study: Smart and Bateman (1967) reported that they had been able to trace reports of 225 adverse reactions to LSD, of which 142 took the form of prolonged psychotic reactions. However, how this number was arrived at was not explained by the authors, and it seems probable that they included sources beyond case reports, e.g. counting patients who had participated in clinical studies of LSD psychosis. One possible reason for the relative paucity of case reports we found is that we were strict about excluding cases where other drugs (apart from cannabis) were also used. Another could be that interest in psychosis as a complication of psychedelic use was at its highest at a time when the concept of schizophrenia was broad, i.e. before the introduction of DSM-III in 1980. However, it has to be said we did not have to exclude many cases for this latter reason.

As noted in the introduction, a longstanding view has been that affective disorder is not a significant complication of psychedelic drug use. When such states have been commented on, they have mostly been considered to take the form of depression, with one review documenting only a single case of a ‘motor excitatory’ state (Smart & Bateman, 1967). In contrast we found as many case reports of affective disorder as of schizophrenia spectrum disorder. Such a finding is arguably not unexpected, given that psychedelics exert their effect on the serotonin system, which has been implicated, and continues to be implicated, in depression for over 50 years (Jauhar, Cowen, & Browning, 2023). Serotonin has also been implicated in bipolar disorder, albeit with less consistent findings (Mahmood & Silverstone, 2001), and with little investigation in recent years. Nevertheless, the ‘bidirectionality’ of the association, i.e. the existence of case reports of both depression and mania after psychedelic drug use, goes against any simplistic conceptualization of the link with serotonin, if there is one.

Could it be that the major psychiatric association of psychedelic drug use is actually with affective disorder, with cases of schizophrenia spectrum disorder being largely or wholly the result of misdiagnosis? While in some ways attractive, this is probably not a tenable position, for two reasons. Firstly, while affective symptoms were present in a proportion of the schizophrenia spectrum cases we found, there were other cases where no affective component was discernible from the descriptions given. Secondly, psychedelic drugs are capable of inducing psychotic symptoms during intoxication itself. Thus, Klee and Weintraub (1959) reported four healthy volunteers who developed sometimes quite elaborate persecutory and referential delusions, and in one case auditory hallucinations, when given LSD under experimental conditions. There is also contemporary evidence to the same effect: Griffiths et al. (2011) described the occurrence of delusions or paranoid thinking in 8 of 18 volunteers while taking psilocybin; examples included believing that a person close to the participant had died, or that the experimenters were malevolently manipulating them. It also seems probable that at least some of the (fortunately rare) cases of individuals who jumped to their deaths, otherwise harmed themselves or committed murder while taking psychedelic drugs (e.g. Asselborn, Wennig, & Yegles, 2000; Blacha et al., 2013; Le Dare, Gicquel, Baert, Morel, & Bouvet, 2020; Reich & Hepps, 1972) did so in response to delusions and/or hallucinations.

Two contemporary population surveys (Johansen & Krebs, 2015; Krebs & Johansen, 2013) found no evidence that recreational use of psychedelics was associated with increased rates of poor mental health, as indexed in various ways. Similarly, a 2020 meta-analysis of 24 studies that examined post-acute effects of psilocybin, ayahuasca and LSD given in clinical or experimental settings (Goldberg et al., 2020) found no evidence of any more than transient adverse effects. Our findings, whilst not directly comparable in terms of methodology, paint a different picture: psychedelic drugs, even when taken only one or a few times, can be followed by major psychiatric adverse events. A recent study using a different methodology from ours also had findings that could be seen as pointing in the same direction. Bremler, Katati, Shergill, Erritzoe, and Carhart-Harris (2023) advertised on social media for individuals who had experienced negative psychological effects after taking psychedelics or MDMA lasting for longer than 72 hours. Those who reported the most severe adverse effects were then invited for an in-person interview. Fifteen participants took part in this second phase, of whom seven took psychedelics only (i.e. not MDMA), and had no previous history of major psychiatric illness. The symptoms they described included anxiety/panic, depression, loss of the ability to experience pleasure, suicidal thoughts, intrusive or obsessive thoughts and behaviors, as well as paranoia/delusional thinking, derealization, distractibility and elevated mood in one case. Two users were diagnosed with bipolar disorder after taking LSD, although both reported experiencing similar symptoms prior to consumption.

Our review of case reports adds detail to the existing, rather sparse descriptive literature on flashbacks. Notably, the experiences described went beyond the conventional view that they take the form simply of ‘psychedelic’ visual phenomena like geometric patterns, distortions and afterimages, but instead could sometimes include formed and sometimes complex images, as well as auditory and occasionally somatosensory experiences. Our findings here are in line with those of Vis, Goudriaan, Ter Meulen, and Blom (2021) who systematically reviewed case reports of HPPD/flashbacks, employing a similar but broader strategy than ours, including MDMA- and cannabis-related experiences and not excluding cases where there was comorbid major psychiatric illness or a history of other drug use. They found formed visual hallucinations, such as faces, skulls, and animals in 9.3% of cases.

We additionally found that flashbacks were not only a consequence of prolonged use of psychedelics but, as with psychosis and major affective disorder, could sometimes develop after taking such drugs only one or a few times. This finding contrasts with those of a questionnaire survey of healthy subjects who took psilocybin between 1 and 4 times as part of a series of experimental studies (Studerus, Kometer, Hasler, & Vollenweider, 2011), which found that only 1 of 90 participants reported the occurrence of flashback-like visual phenomena, and that these were infrequent, brief, and ceased within three days after taking the drug. Rates were higher in another study (Muller et al., 2022), which found that 13 of 142 healthy volunteers who took part in clinical trials of LSD or psilocybin and who had used illicit drugs apart from cannabis no more than 10 times reported flashbacks after participating. The experiences were mostly pleasant or neutral in this study.

The case report literature supports the view that flashbacks are commonly associated with psychiatric symptomatology, and this mostly takes the form of anxiety and depression. Depression, in particular, could sometimes become persistent. Anxiety could accompany the flashbacks themselves, but sometimes extended beyond them to a fear of developing an attack. Our findings remain open on the issue of whether flashbacks are ever ‘benign’, i.e. that they occur without the distress or interference with functioning that would lead to a diagnosis of HPPD: we found 11 out of 29 case reports where no associated psychiatric symptomatology was reported (and one more where the only symptom was worry). However, there are grounds for suspecting that relevant psychiatric description was simply lacking in some of these cases.

In conclusion, this systematic review of case studies supports the view that both psychosis and major affective disorder subsequent to psychedelic drug use exist, highlighting a potential risk in the therapeutic application of these drugs if they are ever approved, and more generally in recreational use. The major limitation of a review of this type is that, while it can say something about the nature of the individual psychiatric complications encountered, it cannot establish rates for their occurrence. Another limitation is that we were unable to apply diagnostic criteria to the case material; because of the wide variation in the richness of the clinical detail provided, this would have resulted in an unacceptably high number of unclassifiable diagnoses. Finally, due to the vagaries of reporting, it is possible that some of the cases included may have been in individuals who used other drugs besides psychedelics. Social and psychological factors contributing to the emergence of psychiatric disorders in response to psychedelic use would also be likely to have been under-reported, if they were reported at all.

## Supporting information

Yildirim et al. supplementary material Yildirim et al. supplementary material

## Supplementary material

The supplementary material for this article can be found at https://doi.org/10.1017/S0033291724002496.
