# Supplementary material for: Adverse psychiatric effects of psychedelic drugs: a systematic review of case reports
Source: Psychol Med. 2024 Nov 20;54(15):4035–47. doi: 10.1017/S0033291724002496 (PMC11650183; doi:10.1017/S0033291724002496)
Supplement: Yildirim et al. supplementary material [file S0033291724002496sup001.docx]

**Supplementary Information for Yildirim et al: Adverse psychiatric effects of psychedelic drugs: a systematic review of case reports.**

*1. Search strategies*

PUBMED search strategy 1

Search was performed 26 June 2023.

(((((((((Hallucinogens[Mesh] OR "Lysergic Acid Diethylamide"[Mesh] OR Psilocybin[Mesh] OR "N,N-Dimethyltryptamine"[Mesh] OR Mescaline[Mesh] OR "Lysergic-Acid-Diethylamide"[tiab] OR LSD[ti] OR Psilocybin*[tiab] OR banisteriopsis[Mesh] OR Ayahuasca[tiab] OR dimethyltryptamine[tiab] OR DMT[ti] OR "5-MeO-DMT"[tiab] OR psychedelic[ti] OR ‘’5-methoxy-N,N-diisopropyltryptamine’’[tiab] OR hallucinogen*[ti] OR peyote[tiab] OR bufotenine[tiab] OR psilocin[tiab]) NOT (Cannabis[Mesh])) NOT (cannab*[ti])) NOT (ibogaine[ti])) NOT (Ibogaine[Mesh])) NOT ("N-Methyl-3,4-methylenedioxyamphetamine"[Mesh])) NOT ("N-Methyl-3,4-methylenedioxyamphetamine"[ti])) NOT (Ecstasy[ti])) NOT (MDMA[ti])

Results: 7793

Filter: case reports

Results: 396

PUBMED search strategy 2

Search was performed 21 June 2023.

((((((((((Hallucinogens[Mesh] OR "Lysergic Acid Diethylamide"[Mesh] OR Psilocybin[Mesh] OR "N,N-Dimethyltryptamine"[Mesh] OR Mescaline[Mesh] OR "Lysergic-Acid-Diethylamide"[tiab] OR LSD[ti] OR Psilocybin*[tiab] OR banisteriopsis[Mesh] OR Ayahuasca[tiab] OR dimethyltryptamine[tiab] OR DMT[ti] OR "5-MeO-DMT"[tiab] OR psychedelic[ti] OR ‘5-methoxy-N,N-diisopropyltryptamine’[tiab] OR hallucinogen*[ti] OR peyote[tiab] OR bufotenine[tiab] OR psilocin[tiab]) NOT (Cannabis[Mesh])) NOT (cannab*[ti])) NOT (ibogaine[ti])) NOT (Ibogaine[Mesh])) NOT ("N-Methyl-3,4-methylenedioxyamphetamine"[Mesh])) NOT ("N-Methyl-3,4-methylenedioxyamphetamine"[ti])) NOT (Ecstasy[ti])) NOT (MDMA[ti])) AND ("Qualitative Research"[Mesh] OR qualitative*[tiab] OR "qualitative research*"[tiab] OR "Focus Groups"[Mesh] OR "focus group*"[tiab] OR "Interviews as Topic"[Mesh] OR interview*[tiab] OR "patient experience*"[tiab] OR "participant experience*"[tiab] OR "subjective experience*"[tiab] OR "interpretative phenomenological analys*"[tiab] OR ‘phenomenolog*[tiab] OR "thematic analys*"[tiab] OR "Grounded Theory"[Mesh] OR "grounded theor*"[tiab] OR "Case Reports" [Publication Type] OR "case report*"[tiab] OR "case-stud*" OR "case histor*"[tiab])

Results: 855

Total Pubmed Search Results: 893

PsyInfo search

Search was performed 30 June 2023 via OVID.

(DE "Hallucinogenic Drugs" OR DE "Bufotenine" OR DE "Lysergic Acid Diethylamide" OR DE "Mescaline" OR DE "Peyote" OR DE "Psilocybin" OR TI (“hallucinogen*” OR “bufotenine” OR “lysergic acid diethylamide” OR “mescaline” OR “peyote” OR “psilocybin” OR “banisteriopsis” OR “N,N-dimethyltryptamine” OR “LSD” OR “ayahuasca” OR “dimethyltryptamine” OR “DMT OR “5-MeO-DMT” OR “psychedelic” OR “hallucinogen*” OR ‘‘5-Methoxy-N,N-diisopropyltryptamine’’) OR AB (“hallucinogen*” OR “bufotenine” OR “lysergic acid diethylamide” OR “mescaline” OR “peyote” OR “psilocybin” OR “banisteriopsis” OR “N,N-dimethyltryptamine” OR “ayahuasca” OR “dimethyltryptamine” OR “5-MeO-DMT” OR “5-Methoxy-N,N-diisopropyltryptamine”)

AND

("Qualitative Methods" OR DE "Focus Group" OR DE "Grounded Theory" OR DE "Interpretative Phenomenological Analysis" OR DE "Narrative Analysis" OR DE "Semi-Structured Interview" OR DE "Thematic Analysis" OR DE "Grounded Theory" OR DE "Case Report" OR TI (“qualitative” OR “focus group*” OR “grounded theor*” OR “interpretative phenomenological analys*” OR “narrative analys*” OR “semi-structured interview” OR “thematic analys*” OR “interview*” OR “patient experience*” OR “participant experience*” OR “subjective experience*” OR “interpretative phenomenological analys*” OR ‘phenomenolog*” OR “grounded theor*” OR “case report*” OR “case-stud*” OR “case histor* OR AB (“controlled stud*” OR “clinical-stud*”OR “qualitative” OR “focus group*” OR “grounded theor*” OR ïnterpretative phenomenological analys*” OR “narrative analys*” OR “semi-structured interview” OR “thematic analys*” OR “interview*” OR “patient experience*” OR “participant experience*” OR “subjective experience*” OR “interpretative phenomenological analys*” OR ‘phenomenolog*” OR “grounded theor*” OR “case report*” OR case-stud*” OR “case histor*)

NOT
(PO Animal NOT PO Human)

Results: 487

Embase

Search was performed 30 June 2023 via OVID.

Embase <1974 to 2023 June 30>

1 *lysergide/ 3831

2 *lysergic acid/ 186

3 *psilocybine/ 1183

4 *ayahuasca/ 165

5 *Banisteriopsis/ 42

6 *n,n dimethyltryptamine/ 548

7 *mescaline/ 897

8 *n,n dimethyltryptamine/ 548

9 *5 methoxy n,n dimethyltryptamine/ 618

10 *psychedelic agent/ 4138

11 hallucinogen*.mp. [mp=title, abstract, heading word, drug trade name, original title, device manufacturer, drug manufacturer, device trade name, keyword heading word, floating subheading word, candidate term word] 5117

12 "peyote".ti,ab. 120

13 "halucinogen*".ti,ab. 5

14 *bufotenine/ 300

15 1 or 2 or 3 or 4 or 5 or 6 or 7 or 8 or 9 or 10 or 11 or 12 or 13 or 14 12922

16 *case study/ 22727

17 *case report/ 22569

18 *qualitative research/ 19664

19 "focus group".ti,ab. 42723

20 *semi structured interview/ 1192

21 *interview/ 9971

22 *personal experience/ 11182

23 *phenomenology/ 2265

24 "patient experience".ab,ti. 16246

25 "subjective experience".ab,ti. 5760

26 "interpretative phenomenological analysis".mp. 3056

27 "case report*".ti,ab. 635939

28 "case stud*".ti,ab. 163515

29 16 or 17 or 18 or 19 or 20 or 21 or 22 or 23 or 24 or 25 or 26 or 27 or 28 914710

30 15 and 29 463

31 limit 30 to human 404

*2. Cases excluded after full text obtained*

Abert et al (2010)^1^

- Multiple exclusions

Abraham (1993)^2^

- Lack of detail

Abraham (1984)^3^

- No use of psychedelics

Abraham & Aldridge (1993)^4^.

- Review article, no cases reported

Abramson 1967^5^

- Book, contains four case reports, but all with insufficient information and/or previous psychiatric history

Alarcon et al (1982)^6^

- Patient described as ‘smoking mushrooms’, also refers to other drug use

Aldurra and Crayton (2001)^7^

- Other drug use

Allen et al (1991)^8^

- Review article, no cases reported

Al Shaban et al (2015)^9^

- Other drug use

Ajithakumari & Hemavathy ((2016)^10^

- No use of psychedelics

Alderliefste (2016)^11^

- Other drug use

Anderson et al (2018)^12^

- Previous psychiatric history

Avery (1970)^13^

- No case reported

Asselborn et al (2000)^14^

- Acute intoxication only

Barber et al (2022)^15^

- Previous psychiatric history

Barbic et al (2020)^16^

- Acute intoxication only

Barter et al (1969)^17^

- Lack of detail

Bender and Hellerstein (2022)^18^

- Review article, no cases reported

Bergman, R. L. (1971)^19^

- Acute intoxication only

Bewley (1967)^20^

- Other drug use

Blacha et al (2013)^21^

- Acute intoxication only

Blacker et al (1968)^22^

- No cases reported

Blaho et al (1997)^23^

- Acute intoxication only

Boer and Sipprelle (1969)^24^

- Other drug use

Bonson et al (1996)^25^

- No cases reported

Borsutzky et al (2002)^26^

- Other drug use

Bose et al (2021)^27^

- Acute intoxication only

Bowers (1972)^28^

- No case reported

Bowers (1977)^29^

- No cases reported

Bowers et al (1967)^30^

- Case 1: acute intoxication only
- Case 2: previous psychiatric history
- Case 3: not clear that he had any psychiatric symptoms

Bowers et al (1995)^31^

- Multiple exclusions

Bowers and Freedman (1966)

- No use of psychedelics

Bremler et al (2023)^32^

- Lack of detail (clinical material but not in case report format), paper referred to in discussion

Brodrick and Mitchell (2016)^33^

- Multiple exclusions

Brown and Stickgold (1976)^34^

- No use of psychedelics

Buck (1967)^35^

- Acute intoxication only

Cartwright (1966)^36^

- No use of psychedelics

Cerón Tapia et al (2022)^37^

- Previous psychiatric history

Christensen et al (2023)^38^

- Alcohol dependence

Cohen (1966)^39^

- Review article, no cases reported

Cohen and Ditman (1963)^40^

- Review article, no cases reported

Cohen et al (1958)^41^

- Acute intoxication only

Cooper (1955)^42^

- Clınıcal study of flashbacks and other symptoms after taking LSD, but no cases reported

Creighton et al (1991)^43^

- No use of psychedelics

Davies (1979)^44^

- Other drug use

Dawson (2001)^45^

- Acute intoxication only

de Pablo Márquez and Dietl Gómez-Luengo (2017)^46^

- Other drug use

Denber and Merlis (1955)^47^

- Study on patients with schizophrenia

Denson (1967)^48^

- Multiple exclusions

Deshon et al (1952)^49^

- No cases reported

Dewhurst (1980)

- Lack of detail

Dewhurst and Hatrick (1972)^50^

- Review article; lack of detail in single case reported

dos Santos et al (2017)^51^

- Lack of detail

dos Santos et al (2017)^52^

- Review article, lack of detail in single case reported

Dufayet et al (2022)^53^

- Multiple exclusions

Duraković et al (2022)^54^

- Lack of detail

Fetter (2005)^55^

- No use of psychedelics

Fink et al (1966)^56^

- Previous psychiatric history, for which LSD given therapeutically

Fischer (1971)^57^

- Review article, no cases reported

Fisher and Undergleider 1968^58^

- Review article no cases reported

Ford et al (2022)^59^

- Review article, lack of detail in reported cases

Friesen, P. (2022)^60^

- Review article, no cases reported

Frosch et al (1965)^61^

- Acute intoxication only

Fuse-Nagase and Nishikawa (2013)^62^

- Drug not a recognized psychedelic

Gaillard and Borruat (2003)^63^

- Case 1 and 2: no use of psychedelics
- Case 3: Probable other drug use, possible alcohol dependency, history of coma

Gelpke (1981)^64^

- Acute intoxication only

Gertsch and Wood (2003)^65^

- Multiple exclusions

Glickman and Blumenfield (1967)^66^

- No cases reported

Goldberg et al (2020)^67^

- Review and meta-analysis, no cases reported

Goldman et al (2007)^68^

- Previous psychiatric history

Gómez-Sousa et al (2021)^69^

- Lack of detail

Göpel et al (2003)^70^

- Acute intoxication only

Guttman (1972)^71^

- None of three cases reported adverse effects

Hanes (1996)^72^

- Case report of treatment of existing body dysmorphic disorder with psilocybin

Hendin et al (2021)^73^

- Previous psychiatric history

Haden and Woods (2020)^74^

- Acute intoxication only

Halpern (2003)^75^

- Review article, no cases reported

Halpern et al (2018)^76^

- No cases reported

Hermle et al (2013)^77^

- Alcohol dependence

Hermle et al (2012)^78^

- Other drug use

Hermle et al (2015)^79^

- Review article, no cases reported

Hoffman (1984)^80^

- No use of psychedelics

Holland and Passie (2011)^81^

- Book on flashbacks, reviews cases from the literature, but no original cases reported

Iaria et al (2010)^82^

- Other drug use

Ifabumuyi and Jeffries (1976)^83^

- Other drug use

Ingram (1964)^84^

- Acute intoxication only

Johnson and Black (2020)^85^

- Case report of therapeutic effects (in cluster headache)

Juve (1972)^86^

- Lack of detail

Keitner et al (1984)^87^

- No use of psychedelics, plus other drug use.

Klee and Weintraub (1959)^88^

- Acute intoxication only (paper referred to in discussion)

Klepfisz and Racy (1973)^89^

- Multiple exclusions

Knudsen (1964)^90^

- Multiple exclusions

Knuijver et al (2018)^91^

- Use of ibogaine, which is not considered as a psychedelic for this review

Kometer and Vollenweider (2018)^92^

- Review article, no cases reported

Kurtom et al (2019)^93^

- More likely a case of PTSD-related flashbacks

Langs and Barr (1968)^94^

- Group study of volunteers given LSD

Lauterbach et al (2000)^95^

- No use of psychedelics

Le Dare et al (2020)^96^

- Acute intoxication only

Leiviskä Deland et al (2011)^97^

- Other (no reference to psychedelics ?wrongly identified by search)

Lerner et al (1998)^98^

- Other drug use

Lerner et al (2000)^99^

- HPPD treatment study, no case report

Lerner et al (2014)^100^

- Other drug use

Lerner et al (2002)^101^

- Lack of detail

Levi andMille (1990)^102^

- No use of psychedelics

Litjens et al (2014)^103^

- Review article, no cases reported

Luce (1971)^104^

- Other drug use

Marsh (1969)^105^

- Lack of detail

Marta et al (2015)^106^

- Use of ibogaine, which is not considered as a psychedelic for this review

Martinotti et al (2018)^107^

- Review article, no cases reported

Matefy and Krall (1974)^108^

- Group study, no cases reported

Matefy (1973)^109^

- Lack of detail

Matefy et al (1979)^110^

- Group study, no cases reported

Matefy et al (1978)^111^

- Group study, no cases reported

McWilliams and Tuttle (1973)^112^

- Review article, no cases reported

Meyer Karre and Heinrich (2014)^113^

- Acute intoxication only

Miller et al (1992)^114^

- Acute intoxication only

Milman (1967)^115^

- Acute intoxication only

Morehead (1997)^116^

- Other drug use

Muller (1971)^117^

- Lack of detail

Musha et al (1986)^118^

- Acute intoxication only

Nielen et al (2004)^119^

- Other drug use

Neven and Blom (2014)^120^

- Other drug use

Nichols and Grob (2018)^121^

- Review article, no cases reported

Nutting et al (2021)^122^

- Previous psychiatric history

Obaydi (1993)^123^

- No use of psyhedelics

Palma-Álvarez et al (2021)^124^

- Other drug use

Paterson et al (2015)^125^

- Other drug use

Perera et al (1995)^126^

- Previous psychiatric history

Perrine et al (1999)^127^

- No case reported

Reich and Hepps (1972)^128^

- Other drug use

Robbins et al (1967)^129^

- Review article, no cases reported

Rosenblat et al (2023)^130^

- Previous psychiatric history

Rubin-Kahana et al (2021)^131^

- Other drug use

Saidel and Babineau (1976)^132^

- Other drug use

Sami et al (2015^133^

- Other drug use (also head injury)

Sandison et al (1954)^134^

- Acute intoxication only

Satora et al (2005)^135^

- Acute intoxication only

Schatten et al (2020)^136^

- Previous psychiatric history

Scher and Neppe (1989)^137^

- Other drug use (2 cases), alcohol dependence (1 case)

Schetz et al (2022)^138^

- No case reported

Shick and Smith (1970)^139^

- Case 1: potential alternative diagnosis of epileptic seizure
- Case 2: lack of detail

Siegel and West (1975)^140^

- Only one case reported, same as Siegel (1992)

Siegel (1992)^141^

- Previous psychiatric history

Smart and Bateman (1967)^142^

- Review article, no cases reported

Smith et al (1980)^143^

- No use of psychedelics

Snyder and Hollister (1967)^144^

- No case reported

Stanciu and Penders (2016)^145^

- Previous psychiatric history

Stoller et al (2017)^146^

- Use of ‘2C’ drug, which are not considered as psychedelics for this review; also acute intoxication only

Strassman (1984)^147^

- Review article, no cases reported

Studerus et al (2011)^148^

- Pooled analysis of group data, no cases reported

Subramanian and Doran (2014)^149^

- Other drug use

Suinn and Brittain (1970)^150^

- Acute intoxication only

Sunness (2004)^151^

- Other drug use

Surawicz and Banta (1975)^152^

- Other drug use

Suzuki (2014)^153^

- Use of 25I-NBOMe, not included in this review (though is a hallucinogen); also acute intoxication only

Szmulewicz et al (2015)^154^

- Previous psychiatric history

Thale et al (1950)^155^

- Study of patients with schizophrenia

Thurlow and Girvin (1971)^156^

- Other drug use

Umut et al (2011)^157^

- Previous psychiatric history

Ungerleider et al (1966)^158^

- Group study, no cases reported

van den Berg et al (2020)^159^

- No case reported

Vardy and Kay (1983)^160^

- Group study, no cases reported

Vis et al (2021)^161^

- Systematic review, no cases reported

Wadsworth (1972)^162^

- Lack of detail

Warren et al (2012)^163^

- Other drug use

Wesson and Smith (1976)^164^

- Review article, no cases reported

Woody (1970)^165^

- Other drug use

Yeniocak et al (2019)^166^

- Acute intoxication only

1. Abert B, Ilsen PF. Palinopsia. *Optometry* 2010; **81**: 394-404.

2. Abraham HD. L-5-hydroxytryptophan for LSD-induced psychosis. *Am J Psychiatry* 1983; **140**: 456-8.

3. Abraham HD. LSD flashbacks (letter). *Arch Gen Psychiatry* 1984; **41**: 632-3.

4. Abraham HD, Aldridge AM. Adverse consequences of lysergic acid diethylamide. *Addiction* 1993; **88**: 1327-34.

5. Abramson HA. The use of LSD in psychotherapy and alcoholism. Indianapolis,: Bobbs-Merrill; 1967.

6. Alarcon RD, Dickinson WA, Dohn HH. Flashback phenomena. Clinical and diagnostic dilemmas. *J Nerv Ment Dis* 1982; **170**: 217-23.

7. Aldurra G, Crayton JW. Improvement of hallucinogen persisting perception disorder by treatment with a combination of fluoxetine and olanzapine: case report. *J Clin Psychopharmacol* 2001; **21**: 343-4.

8. Allen JW, Merlin MD, Jansen KL. An ethnomycological review of psychoactive agarics in Australia and New Zealand. *J Psychoactive Drugs* 1991; **23**: 39-69.

9. Al Shaban OWMA, Álvarez de Morales Gómez-Moreno E, López-Fernández JR. Trastorno perceptivo persistente por alucinógenos: a propósito de un caso. *Psiquiatría Biológica* 2015; **22**: 56-8.

10. Ajithakumari G, Hemavathy V. Alice in Wonderland: A case study. *Res J Pharmaceut, Biol Chem Sci* 2016; **7**: 217-21.

11. Alderliefste GJ. DPS en HPPD: signalering, diagnostiek en behandeling van persistente waarnemingsstoornissen na partydrugs. *Verslaving* 2016; **12** 172-84.

12. Anderson L, Lake H, Walterfang M. The trip of a lifetime: hallucinogen persisting perceptual disorder. *Australas Psychiatry* 2018; **26**: 11-2.

13. Avery TL. L.S.D. in psychiatry. *Lancet* 1970; **296(7685)**: 1251.

14. Asselborn G, Wennig R, Yegles M. Tragic flying attempt under the influence of "magic mushrooms". *Problems Forensic Sc* 2000; **XLII**: 41-6.

15. Barber G, Nemeroff CB, Siegel S. A Case of Prolonged Mania, Psychosis, and Severe Depression After Psilocybin Use: Implications of increased psychedelic drug availability. *Am J Psychiatry* 2022; **179**: 892-6.

16. Barbic D, Fernandes J, Eberdt C, Chakraborty A. N,N-Dimethyltryptamine: DMT-induced psychosis. *Am J Emerg Med* 2020; **38**: 1961 e1- e2.

17. Barter JT, Reite M. Crime and LSD: the insanity plea. *Am J Psychiatry* 1969; **126**: 531-7.

18. Bender D, Hellerstein DJ. Assessing the risk-benefit profile of classical psychedelics: a clinical review of second-wave psychedelic research. *Psychopharmacology (Berl)* 2022; **239**: 1907-32.

19. Bergman RL. Navajo peyote use: its apparent safety. *Am J Psychiatry* 1971; **128**: 695-9.

20. Bewley TH. Adverse reactions from the illicit use of lysergide. *Br Med J* 1967; **3**: 28-30.

21. Blacha C, Schmid MM, Gahr M, et al. Self-inflicted testicular amputation in first lysergic acid diethylamide use. *J Addict Med* 2013; **7**: 83-4.

22. Blacker KH, Jones RT, Stone GC, Pfefferbaum D. Chronic users of LSD: the "acidheads". *Am J Psychiatry* 1968; **125**: 97-107.

23. Blaho K, Merigian K, Winbery S, Geraci SA, Smartt C. Clinical pharmacology of lysergic acid diethylamide: case reports and review of the treatment of intoxication. *Am J Ther* 1997; **4**: 211-21.

24. Boer AP, Sipprelle CN. Induced anxiety in the treatment for LSD effects. *Psychother Psychosom* 1969; **17**: 108-13.

25. Bonson KR, Buckholtz JW, Murphy DL. Chronic administration of serotonergic antidepressants attenuates the subjective effects of LSD in humans. *Neuropsychopharmacology* 1996; **14**: 425-36.

26. Borsutzky M, Passie T, Paetzold, W., Emrich HM, Schneider U. [Hawaiian baby woodrose: (Psycho-) Pharmacological effects of the seeds of Argyreia nervosa. A case-orientated demonstration]. *Nervenarzt* 2002; **73,**: 892-6.

27. Bose PK, Ray D, Biswas P, Arafat SMY. Suicidal cut-throat wound during LSD intoxication. *Clin Case Rep* 2021; **9**: e05100.

28. Bowers MB, Jr. Acute psychosis induced by psychotomimetic drug abuse. I. Clinical findings. *Arch Gen Psychiatry* 1972; **27**: 437-40.

29. Bowers MB, Jr. Psychoses precipitated by psychotomimetic drugs. A follow-up study. *Arch Gen Psychiatry* 1977; **34**: 832-5.

30. Bowers M, Chipman A, Schwartz A, Dann OT. Dynamics of psychedelic drug abuse. A clinical study. *Arch Gen Psychiatry* 1967; **16**: 560-6.

31. Bowers MB, Jr., Imirowicz R, Druss B, Mazure CM. Autonomous psychosis following psychotogenic substance abuse. *Biol Psychiatry* 1995; **37**: 136-7.

32. Bremler R, Katati N, Shergill P, Erritzoe D, Carhart-Harris RL. Case analysis of long-term negative psychological responses to psychedelics. *Sci Rep* 2023; **13**: 15998.

33. Brodrick J, Mitchell BG. Hallucinogen persisting perception disorder and risk of suicide. *J Pharm Pract* 2016; **29**: 431-4.

34. Brown A, Stickgold A. Marijuana flashback phenomena. *J Psychedelic Drugs,* 1976; **8**: 275-83.

35. Buck RW. Psychedelic effect of Pholiota spectabilis. *N Eng J Med* 1967; **276**: 391-2.

36. Cartwright RD. Dream and drug-induced fantasy behavior. A comparative study. *Arch Gen Psychiatry* 1966; **15**: 7-15.

37. Cerón Tapia HR, González Guzmán MA, Córdoba Ortiz SA. Ayahuasca-induced psychosis: A case report. *Revista Colombiana de psiquiatria* 2022; **51**: 236-9.

38. Christensen JA, Fipps DC, Bostwick JM. To treat or not to treat? High-potency benzodiazepine use in a case of comorbid hallucinogen persisting perception disorder and alcohol use disorder. *Exp Clin Psychopharmacol* 2023; **31**: 300-4.

39. Cohen S. A classification of LSD complications. *Psychosomatics* 1966; **7**: 182-6.

40. Cohen S, Ditman KS. Prolonged adverse reactions to lysergic acid diethylamide. *Arch Gen Psychiatry* 1963; **8**: 475-80.

41. Cohen S, Fichman L, Grover Eisner B. Subjective reports of lysergic acid experiences in a context of psychological test performance. *Am J Psychiatry* 1958; **115**: 30-5.

42. Cooper HA. Hallucinogenic drugs. *Lancet* 1955; **268**: 1078-9.

43. Creighton FJ, Black DL, Hyde CE. 'Ecstasy' psychosis and flashbacks. *Br J Psychiatry* 1991; **159**: 713-5.

44. Davies NS. Psychiatric symptoms and hallucinogenic compounds. *Br Med J* 1979; **2**: 797.

45. Dawson KA. A case study of space-time distortion during a total lunar eclipse following street use of LSD. *J Psychoactive Drugs* 2001; **33**: 301-5.

46. de Pablo Márquez B, Dietl Gómez-Luengo B. Ayahuasca intoxication. *Medicina Clinica* 2017; **149**: 136-7.

47. Denber HC, Merlis S. Studies on mescaline. I. Action in schizophrenic patients; clinical observations and brain wave patterns, showing effects before and after electric convulsive treatments. *Psychiatr Q* 1955; **29**: 421-9.

48. Denson R. Dissociative delirium after treatment with lysergide. *Can Med Assoc J* 1967; **97**: 1222-4.

49. DeShon HJ, Rinkel M, Solomon HC. Mental changes experimentally produced by d-lysergic acid diethylamide tartrate. *Psychiatr Q* 1952; **26**: 33-53.

50. Dewhurst K, Hatrick JA. Differential diagnosis and treatment of lysergic acid diethylamide induced psychosis. *Practitioner* 1972; **209**: 327-32.

51. Dos Santos RG, Osório FL, Crippa JAS, Hallak JE. Anxiety, panic, and hopelessness during and after ritual ayahuasca intake in a woman with generalized anxiety disorder: a case report. *J psychedelic Studies* 2017; **1** 35-9.

52. Dos Santos RG, Bouso JC, Hallak JEC. Ayahuasca, dimethyltryptamine, and psychosis: a systematic review of human studies. *Ther Adv Psychopharmacol* 2017; **7**: 141-57.

53. Dufayet L, Langrand J, Alvarez JC, Islam Larabi A. Loss of Consciousness and Visual Hallucinations Related to 5-MeO-DALT Intake, a Case Report Confirmed by Toxicological Analyses. *J Anal Toxicol* 2022; **46**: e186-e90.

54. Duraković D, Silić A, Vjekoslav P, Vlahović D, Vojnović D, Karlović D. Acute mescaline intoxication followed by catatonia *Arch Psychiatr Res* 2022; **58**: 287-92.

55. Fetter JC. Mirtazepine for MDMA-induced depression. *Am J Addict* 2005; **14**: 300-1.

56. Fink M, Simeon J, Haque W, Itil T. Prolonged adverse reactions to LSD in psychotic subjects. *Arch Gen Psychiatry* 1966; **15**: 450-4.

57. Fischer R. The “flashback”: arousal-statebound recall of experience. *J Psychedelic Drugs* 1971 **3**

58. Fisher DD, Ungerleider JT. The therapy of untoward LSD reactions. *Curr Psychiatr Ther* 1968; **8** 110-4.

59. Ford H, Fraser CL, Solly E, et al. Hallucinogenic Persisting Perception Disorder: A Case Series and Review of the Literature. *Front Neurol* 2022; **13**: 878609.

60. Friesen P. Psychosis and psychedelics: Historical entanglements and contemporary contrasts. *Transcult Psychiatry* 2022; **59**: 592-609.

61. Frosch WA, Robbins ES, Stern M. Untoward reactions to lysergic acid diethylamide (LSD) resulting in hospitalization. *N Engl J Med* 1965; **273**: 1235-9.

62. Fuse-Nagase Y, Nishikawa T. Prolonged delusional state triggered by repeated ingestion of aromatic liquid in a past 5-methoxy-N, N-diisopropyltryptamine abuser. *Addict Sci Clin Pract* 2013; **8**: 9.

63. Gaillard M-C, Borruat F-X. Persisting visual hallucinations and illusions in previously drug-addicted patients. *Klinische Monatsblatter Fur Augenheilkunde* 2003; **220**: 176-8.

64. Gelpke R. On travels in the universe of the soul: reports on self-experiments with delysid (LSD) and psilocybin (CY). *J Psychoactive Drugs* 1981; **13**: 81-9.

65. Gertsch JH, Wood C. Case report: An ingestion of Hawaiian baby woodrose seeds associated with acute psychosis. *Hawaii Med J* 2003; **62** 127-9.

66. Glickman L, Blumenfield M. Psychological determinants of "LSD reactions". *J Nerv Ment Dis* 1967; **145**: 79-83.

67. Goldberg SB, Shechet B, Nicholas CR, et al. Post-acute psychological effects of classical serotonergic psychedelics: a systematic review and meta-analysis. *Psychol Med* 2020; **50**: 2655-66.

68. Goldman S, Galarneau D, Friedman R. New Onset LSD Flashback Syndrome Triggered by the Initiation of SSRIs. *Ochsner J* 2007; **7**: 37-9.

69. Gomez-Sousa M, Jimenez-Garrido DF, Ona G, et al. Acute Psychological Adverse Reactions in First-Time Ritual Ayahuasca Users: A Prospective Case Series. *J Clin Psychopharmacol* 2021; **41**: 163-71.

70. Göpel C, Maras A, Schmidt MH. [Hawaiian baby rose wood: case report of an argyreia nervosa induced toxic psychosis]. *Psychiatr Prax* 2003; **30**: 223-4.

71. Guttman HA. The first trip: life crisis and the first experience with hallucinogenic drugs. *J Nerv Ment Dis* 1972; **154**: 453-6.

72. Hanes KR. Serotonin, psilocybin, and body dysmorphic disorder: a case report. *J Clin Psychopharmacol* 1996; **16**: 188-9.

73. Hendin HM, Penn AD. An episode of mania following self-reported ingestion of psilocybin mushrooms in a woman previously not diagnosed with bipolar disorder: A case report. *Bipolar Disord* 2021; **23**: 733-5.

74. Haden M, Woods B. LSD Overdoses: Three Case Reports. *J Stud Alcohol Drugs* 2020; **81**: 115-8.

75. Halpern JH. Hallucinogens: an update *Curr Psychiatr Rep* 2003; **5,** : 347-54.

76. Halpern JH, Lerner AG, Passie T. A Review of Hallucinogen Persisting Perception Disorder (HPPD) and an Exploratory Study of Subjects Claiming Symptoms of HPPD. *Curr Top Behav Neurosci* 2018; **36**: 333-60.

77. Hermle L, Simon M, Ruchsow M, Batra A, Geppert M. Hallucinogen Persisting Perception Disorder (HPPD) and Flashback-are they Identical? *J Alcohol Drug Depend* 2013; **1**: 1-4.

78. Hermle L, Simon M, Ruchsow M, Geppert M. Hallucinogen-persisting perception disorder. *Ther Adv Psychopharmacol* 2012; **2**: 199-205.

79. Hermle L, Ruchsow M, Taschner KL. [Hallucinogen persisting perception disorder (hppd) and flashback phenomena - differential diagnosis and explanation models]. *Fortschr Neurol Psychiatr* 2015; **83**: 506-15.

80. Hoffman JA. LSD flashbacks. *Arch Gen Psychiatry* 1984; **41**: 631-2.

81. Holland D, Passie T. Flashback-Phänomene: als Nachwirkng von Halluzinogeneinnahme. Berlin: VWB-Verlag für Wissenschaft und Bildung; 2011.

82. Iaria G, Fox CJ, Scheel M, Stowe RM, Barton JJ. A case of persistent visual hallucinations of faces following LSD abuse: a functional Magnetic Resonance Imaging study. *Neurocase* 2010; **16**: 106-18.

83. Ifabumuyi OI, Jeffries JJ. Treatment of drug-induced psychosis with diphenylhydantoin *Can Psychiatr Assoc J* 1976; **21**: 565-9.

84. Ingram AL, Jr. Morning Glory Seed Reaction. *JAMA* 1964; **190**: 1133-4.

85. Johnson S, Black QC. Can Psychedelics Alleviate Symptoms of Cluster Headache and Accompanying Mental Health Problems? A Case Report Involving Hawaiian Baby Woodrose. *J Psychoactive Drugs* 2020; **52**: 319-23.

86. Juve JL. Bad drug trips and flashbacks. *Child Welfare* 1972; **51**: 41-50.

87. Keitner GI, Sabaawi M, Haier RJ. Isosafrole and schizophrenia-like psychosis. *Am J Psychiatry* 1984; **141**: 997-8.

88. Klee GD, Weintraub W. Paranoid reactions following lysergic acid diethylamide (LSD-25). In: Bradley PB, Deniker P, Radouco-Thomas C, eds. Neuro-psychopharmacology: proceedings of the first international congress of neuro-psychopharmacology, Rome, September 1958. Amsterdam: Elsevier; 1959: 457-60.

89. Klepfisz A, Racy J. Homicide and LSD. *JAMA* 1973; **223**: 429-30.

90. Knudsen K. Homicide after Treatment with Lysergic Acid Diethylamide. *Acta Psychiatr Scand* 1964; **39**: 389-95.

91. Knuijver T, Belgers M, Markus W, Verkes RJ, van Oosteren T, Schellekens A. Hallucinogen persisting perception disorder after ibogaine treatment for opioid dependence. *J Clin Psychopharmacol* 2018; **38**: 646-8.

92. Kometer M, Vollenweider FX. Serotonergic Hallucinogen-Induced Visual Perceptual Alterations. *Curr Top Behav Neurosci* 2018; **36**: 257-82.

93. Kurtom M, Henning A, Espiridion ED. Hallucinogen-persisting Perception Disorder in a 21-year-old Man. *Cureus* 2019; **11**: e4077.

94. Langs RJ, Barr HL. Lysergic acid diethylamide (LSD-25) and schizophrenic reactions. A comparative study. *J Nerv Ment Dis* 1968; **147**: 163-72.

95. Lauterbach EC, Abdelhamid A, Annandale JB. Posthallucinogen-like visual illusions (palinopsia) with risperidone in a patient without previous hallucinogen exposure: possible relation to serotonin 5HT2a receptor blockade. *Pharmacopsychiatry* 2000; **33**: 38-41.

96. Le Dare B, Gicquel T, Baert A, Morel I, Bouvet R. Self-inflicted neck wounds under influence of lysergic acid diethylamide: A case report and literature review. *Medicine (Balt)* 2020; **99**: e20868.

97. Leiviskä Deland AC, Karlsson G, Fatouros-Bergman H. A phenomenological analysis of the psychotic experience *Human Studies* 2011; **34**: 23-42.

98. Lerner AG, Finkel B, Oyffe I, Merenzon I, Sigal M. Clonidine treatment for hallucinogen persisting perception disorder. *Am J Psychiatry* 1998; **155**: 1460.

99. Lerner AG, Gelkopf M, Oyffe I, et al. LSD-induced hallucinogen persisting perception disorder treatment with clonidine: an open pilot study. *Int Clin Psychopharmacol* 2000; **15**: 35-7.

100. Lerner AG, Rudinski D, Lev-Ran S. LSD flashbacks-the appearance of new visual imagery not experienced during initial intoxication: two case reports. *Israel J Psychiatry* 2014; **51** 307-9.

101. Lerner AG, Shufman E, Kodesh A, Kretzmer G, Sigal M. LSD-induced hallucinogen persisting perception disorder with depressive features treated with reboxetine: case report. *Israel J Psychiatry* 2002; **39**: 100-3.

102. Levi L, Miller NR. Visual illusions associated with previous drug abuse. *J Clin Neuroophthalmol* 1990; **10**: 103-10.

103. Litjens RP, Brunt TM, Alderliefste GJ, Westerink RH. Hallucinogen persisting perception disorder and the serotonergic system: a comprehensive review including new MDMA-related clinical cases. *Eur Neuropsychopharmacol* 2014; **24**: 1309-23.

104. Luce J. The end of the road: A case study. *J Psychedelic Drugs* 1971; **4,**: 50-2.

105. Marsh A. Visual hallucinations during hallucinogenic experience and schizophrenia. *Schizophr Bull* 1979; **5**: 627-30.

106. Marta CJ, Ryan WC, Kopelowicz A, Koek RJ. Mania following use of ibogaine: A case series. *Am J Addict* 2015; **24**: 203-5.

107. Martinotti G, Santacroce R, Pettorruso M, et al. Hallucinogen persisting perception disorder: Etiology, clinical features, and therapeutic perspectives. *Brain Sci* 2018; **8**.

108. Matefy RE, Krall RG. An initial investigation of the psychedelic drug flashback phenomena. *J Consult Clin Psychol* 1974; **42**: 854-60.

109. Matefy RE. Behavior therapy to extinguish spontaneous recurrences of LSD effects: a case study. *J Nerv Ment Dis* 1973; **156**: 226-31.

110. Matefy RE, Hayes C, Hirsch J. Psychedelic drug flashbacks: attentional deficits? *J Abnorm Psychol* 1979; **88**: 212-5.

111. Matefy RE, Hayes C, Hirsch J. Psychedelic drug flashbacks: subjective reports and biographical data. *Addict Behav* 1978; **3**: 165-78.

112. McWilliams SA, Tuttle RJ. Long-term psychological effects of LSD. *Psychol Bull* 1973; **79**: 341-51.

113. Meyer Karre VM, Heinrich T. Ingestion of Hawaiian Baby Woodrose seeds to obtain a legal high. *Psychosomatics* 2014; **55**: 517-8.

114. Miller PL, Gay GR, Ferris KC, Anderson S. Treatment of acute, adverse psychedelic reactions: ‘I’ve tripped and I can’t get down. *J Psychoactive Drugs* 1992; **24**: 272-9.

115. Milman DH. An untoward reaction to accidental ingestion of LSD in a 5-year-old girl. *JAMA* 1967; **201**: 821-5.

116. Morehead DB. Exacerbation of hallucinogen-persisting perception disorder with risperidone. *J Clin Psychopharmacol* 1997; **17**: 327-8.

117. Muller DJ. ECT in LSD psychosis. a report of three cases. *Am J Psychiatry* 1971; **128**: 351-2.

118. Musha M, Ishii A, Tanaka F, Kusano G. Poisoning by hallucinogenic mushroom hikageshibiretake (Psilocybe argentipes K. Yokoyama) indigenous to Japan. *Tohoku J Exp Med* 1986; **148**: 73-8.

119. Nielen RJ, van der Heijden FM, Tuinier S, Verhoeven WM. Khat and mushrooms associated with psychosis. *World J Biol Psychiatry* 2004; **5**: 49-53.

120. Neven A, Blom JD. [Synesthesias in the context of hallucinogen-induced persistent perception disorder following the use of lsd]. *Tijdschr Psychiatr* 2014; **56**: 748-52.

121. Nichols DE, Grob CS. Is LSD toxic? *Forensic Sci Int* 2018; **284**: 141-5.

122. Nutting S, Bruinsma T, Anderson M, Jolly T. Psychotic and still tripping—Hallucinogen persisting perception disorder and first break psychosis in an adolescent *Int J Ment Health Addiction* 2021; **19**: 2440-2.

123. Obaydi H. Flashbacks and schizophrenia *Irish J Psychol Med* 1993; **10**: 37-9.

124. Palma-Alvarez RF, Grau-Lopez L, Ros-Cucurull E, et al. Psychosis induced by abuse of ayahuasca: a case report. *Rev Colomb Psiquiatr (Engl Ed)* 2021; **50**: 43-6.

125. Paterson NE, Darby WC, Sandhu PS. N,N-Dimethyltryptamine-induced psychosis. *Clin Neuropharmacol* 2015; **38**: 141-3.

126. Perera KM, Ferraro A, Pinto MR. Catatonia LSD induced? *Aust N Z J Psychiatry* 1995; **29**: 324-7.

127. Perrine DM. Hallucinogens and obsessive-compulsive disorder. *Am J Psychiatry* 1999; **156**: 1123.

128. Reich P, Hepps RB. Homicide during a psychosis induced by LSD. *JAMA* 1972; **219**: 869-71.

129. Robbins E, Robbins L, Frosch WA, Stern M. Implications of untoward reactions to hallucinogens. *Bull N Y Acad Med* 1967; **43**: 985-99.

130. Rosenblat JD, Leon-Carlyle M, Ali S, Husain MI, McIntyre RS. Antidepressant effects of psilocybin in the absence of psychedelic effects. *Am J Psychiatry* 2023; **180**: 395-6.

131. Rubin-Kahana DS, Hassan AN, Le Foll B. Posttraumatic Stress Disorder After a Psychedelic Experience, a Case Report. *J Addict Med* 2021; **15**: 248-51.

132. Saidel DR, Babineau R. Prolonged LSD flashbacks as conversion reactions. *J Nerv Ment Dis* 1976; **163**: 352-5.

133. Sami M, Piggott K, Coysh C, Fialho A. Psychosis, psychedelic substance misuse and head injury: A case report and 23 year follow-up. *Brain Inj* 2015; **29**: 1383-6.

134. Sandison RA, Spencer AM, Whitelaw JD. The therapeutic value of lysergic acid diethylamide in mental illness. *J Ment Sci* 1954; **100**: 491-507.

135. Satora L, Goszcz H, Ciszowski K. Poisonings resulting from the ingestion of magic mushrooms in Krakow. *Przegl Lek* 2005; **62**: 394-6.

136. Schatten H, Eter N, Mihailovic N. [Visual snow in hallucinogen-persisting perception disorder]. *Ophthalmologe* 2020; **117**: 1112-5.

137. Scher M, Neppe V. Carbamazepine adjunct for nonresponsive psychosis with prior hallucinogenic abuse. *J Nerv Ment Dis* 1989; **177**: 755-7.

138. Schetz D, Schetz A, Kocic I. A retrospective analysis of the "Neverending Trip" after administration of a potent full agonist of 5-HT2A receptor - 25I-NBOMe. *Biomed Pharmacother* 2022; **146**: 112295.

139. Shick JFE, Smith DE. Analysis of the LSD flashback. *J Psychedelic Drugs* 1970; **3**: 13-9.

140. Siegel RK, West LJ. Hallucinations: behavior, experience, and theory. New York: Wiley; 1975.

141. Siegel RK. Fire in the brain: clinical tales of hallucination. New York, N.Y., U.S.A.: Dutton; 1992.

142. Smart RG, Bateman K. Unfavourable reactions to LSD: a review and analysis of the available case reports. *Can Med Assoc J* 1967; **97**: 1214-21.

143. Smith JA, Walters G, Johnston D. LSD 'flashback' as a cause of diagnostic error. *Postgrad Med J* 1980; **56**: 421-2.

144. Snyder SH, Faillace L, Hollister L. 2,5-dimethoxy-4-methyl-amphetamine (STP): a new hallucinogenic drug. *Science* 1967; **158**: 669-70.

145. Stanciu CN, Penders TM. Hallucinogen persistent perception disorder induced by new psychoactive substituted phenethylamines; A review with illustrative case. *Curr Psychiatr Rev* 2016; **12** 221-3.

146. Stoller A, Dolder PC, Bodmer M, et al. Mistaking 2C-P for 2C-B: What a Difference a Letter Makes. *J Anal Toxicol* 2017; **41**: 77-9.

147. Strassman RJ. Adverse reactions to psychedelic drugs. A review of the literature. *J Nerv Ment Dis* 1984; **172**: 577-95.

148. Studerus E, Kometer M, Hasler F, Vollenweider FX. Acute, subacute and long-term subjective effects of psilocybin in healthy humans: a pooled analysis of experimental studies. *J Psychopharmacol* 2011; **25**: 1434-52.

149. Subramanian N, Doran M. Improvement of hallucinogen persisting perception disorder (HPPD) with oral risperidone: case report. *Ir J Psychol Med* 2014; **31**: 47-9.

150. Suinn RM, Brittain J. The termination of an LSD ‘freak-out’ through the use of relaxation. *J Clin Psychology* 1970; **26** 127-8.

151. Sunness JS. Persistent afterimages (palinopsia) and photophobia in a patient with a history of LSD use. *Retina* 2004; **24**: 805.

152. Surawicz FG, Banta R. Lycanthropy revisited. *Can Psychiatr Assoc J* 1975; **20**: 537-42.

153. Suzuki J, Poklis JL, Poklis A. “My friend said it was good LSD”: A suicide attempt following analytically confirmed 25I-NBOMe ingestion. *J Psychoactive Drugs* 2014; **46**: 379-82.

154. Szmulewicz AG, Valerio MP, Smith JM. Switch to mania after ayahuasca consumption in a man with bipolar disorder: a case report. *Int J Bipolar Disord* 2015; **3**: 4.

155. Thale T, Gabrio BW, Salomon K. Hallucination and imagery induced by mescaline. *Am J Psychiatry* 1950; **106**: 686-91.

156. Thurlow HJ, Girvin JP. Use of anti-epileptic medication in treating "flashbacks" from hallucinogenic drugs. *Can Med Assoc J* 1971; **105**: 947-8.

157. Umut G, Küçükparlak İ, Özgen G, Türkcan A. A mood disorder episode with an onset under chronic cannabis consumption and accompanied with psychotic features immediately after N, N-Dimethyltryptamine (DMT) use: case report. *Dusunen Adam Journal of Psychiatry and Neurological Sciences* 2011; **24**: 246-50.

158. Ungerleider JT, Fisher DD, Fuller M. The dangers of LSD. Analysis of seven months' experience in a university hospital's psychiatric service. *JAMA* 1966; **197**: 389-92.

159. van den Berg A, van Eeghen EE, Kooter AJ, Tuinman TH, Tuinman PR. Ayahuasca in de polder Wat een dokter moet weten over de bijwerkingen. *Nederlands Tijdschrift voor Geneeskunde* 2020; **164**: D4501.

160. Vardy MM, Kay SR. LSD psychosis or LSD-induced schizophrenia? A multimethod inquiry. *Arch Gen Psychiatry* 1983; **40**: 877-83.

161. Vis PJ, Goudriaan AE, Ter Meulen BC, Blom JD. On Perception and Consciousness in HPPD: A Systematic Review. *Front Neurosci* 2021; **15**: 675768.

162. Wadsworth ML. Peyote use: a case report. *Am J Psychiatry* 1972; **129**: 96.

163. Warren JM, Dham-Nayyar P, Alexander J. Recreational use of naturally occurring dimethyltryptamine--contributing to psychosis? *Aust N Z J Psychiatry* 2013; **47**: 398-9.

164. Wesson DR, Smith DE. An analysis of psychedelic drug flashbacks. *Am J Drug Alcohol Abuse* 1976; **3**: 425-38.

165. Woody GE. Visual disturbances experienced by hallucinogenic drug abusers while driving. *Am J Psychiatry* 1970; **127**: 683-6.

166. Yeniocak S, Kalkan A, Agus T, Demirel A, Akkoc I, Katipoglu B. A rare case: Ayahuasca tea intoxication. *Akademik Acil Tip Olgu Sunumlari Dergisi* 2019; **10**: 75-7.

*3. Papers not located*

Amurao AA, Duncan WH. LSD ingestion. A review with four case reports. Delaware Medical Journal 1969; Oct;41(10):289-92.

- British Library doesn’t hold for this year

Assmus H, Reimer F. Accidental LSD intoxication of three brothers and sisters in childhood with ‘echo-psychosis’ ('flashback’). Praxis der Kinderpsychologie und Kinderpsychiatrie 1972; 21(6): 207-209.

- British Library doesn’t hold for this year

Blumenfield M, Glickman L. Ten months experience with LSD users admitted to county psychiatric receiving hospital. New York State Journal of Medicine 1967; 67(13):1849-1853

- Not in British Library (relevant issue for that year missing)

Holsten F. Flashbacks. Klinisk og sosial betydning 1 1/2--4 år etter første behandlingskontakt [Flashbacks. Clinical and social significance 1 1/2–4 years after the 1st admission]. Tidsskr Nor Laegeforen 1976; 96(15):875-878.

- British Library doesn’t hold for this year

Kristensen LH, Sørensen BH. Vedvarende symptomer efter indtagelse afhallucinogene svampe [Persistent symptoms after intake of hallucinogenic mushrooms]. Ugeskr Laeger 1988;150(20):1224-5.

- In British Library but not currently obtainable (due to cyberattack)

Lee JWY. Possibility of LSD-induced catatonia. Australian and New Zealand Journal of Psychiatry 1995; 29 (4): 696-97.

- British Library doesn’t hold for this year

MacDonald J, Agar M, Leigh A, Henderson LA, Glass WJ. “What is a trip—and why take one?” LSD: Still with us after all these years. 1998; [no volume number]:9-36. Jossey-Bass.

- Can’t trace journal

Sizaret P, Degiovanni A, Jamin C. “On a case of hallucinogenic delusion”. Annales Médico-Psychologiques 1977; 1(5): 798-801.

- British Library doesn’t hold for this year
